# Modification and Functionalization of Separators for High Performance Lithium–Sulfur Batteries

**DOI:** 10.3390/ijms252111446

**Published:** 2024-10-24

**Authors:** Mengyu Shen, Songshi Xu, Xiuyu Wang, Yonghui Zhang, Yu Feng, Fei Xing, Yingying Yang, Qiqian Gao

**Affiliations:** School of Physics and Optoelectronic Engineering, Shandong University of Technology, Zibo 255000, China; shenmengyu123456@163.com (M.S.); wfxusongshi@126.com (S.X.); wangxiuyu1022@163.com (X.W.); yhzhang@sdut.edu.cn (Y.Z.); fywg@sdut.edu.cn (Y.F.); yangyingying@sdut.edu.cn (Y.Y.)

**Keywords:** lithium–sulfur batteries, functional separator, polysulfide shuttle effect

## Abstract

Lithium–sulfur batteries (LSB) have been recognized as a prominent potential next-generation energy storage system, owing to their substantial theoretical specific capacity (1675 mAh g^−1^) and high energy density (2600 Wh kg^−1^). In addition, sulfur’s abundance, low cost, and environmental friendliness make commercializing LSB feasible. However, challenges such as poor cycling stability and reduced capacity, stemming from the formation and diffusion of lithium polysulfides (LiPSs), hinder LSB’s practical application. Introducing functional separators represents an effective strategy to surmount these obstacles and enhance the electrochemical performance of LSBs. Here, we have conducted a comprehensive review of recent advancements in functional separators for LSBs about various (i) carbon and metal compound materials, (ii) polymer materials, and (iii) novel separators in recent years. The detailed preparation process, morphology and performance characterization, and advantages and disadvantages are summarized, aiming to fundamentally understand the mechanisms of improving battery performance. Additionally, the development potential and future prospects of advanced separators are also discussed.

## 1. Introduction

With the rapid progress in scientific and technological developments in contemporary society, challenges such as energy crises and environmental pollution have emerged as significant concerns. In 2020, fossil fuels like coal, oil, and natural gas accounted for 61.3% of global electricity generation, highlighting the urgent need for clean and renewable energy sources [1,2,3]. While solar and wind energy have been extensively developed, their inherent traits such as dispersion, intermittency, and lack of controllability pose limitations to their further expansion [4]. Energy storage systems play a crucial role in balancing electricity generation and consumption by storing and releasing electrical energy, thus addressing the issue of power imbalance [5]. There is a pressing demand to develop stable and high-capacity clean energy storage systems to meet societal energy needs. Among the diverse range of energy storage solutions, lithium-ion batteries (LIBs) have garnered attention due to their high safety, extended lifespan, and environmental compatibility, finding widespread applications in electric vehicles and aerospace industries [6,7]. However, due to the limitations of the structure of its cathode material, the energy density of LIBs assembled with typical cathode materials is less than 300 Wh kg^−1^ [8], which limits the development of LIBs to a certain extent and leads to the fact that it is also difficult for them to meet the rapid development of electric vehicles and portable electronic devices [9]. Furthermore, the electrochemical performance of LIBs has approached the theoretical limit, resulting in no further development space for LIBs. Therefore, the development of a new secondary battery system has become an inevitable trend.

LSBs have emerged as a focal point for research due to their high theoretical specific capacity (1675 mAh g^−1^) of sulfur and high energy density (2600 Wh kg^−1^), as well as the abundant reserves of elemental sulfur. LSBs stand out as the most prospective candidate in the realm of energy storage solutions owing to their affordability and eco-friendly nature [10,11]. However, during development, LSBs encounter numerous challenges including low electrical conductivity of sulfur, significant volume expansion of the cathode due to the S to Li_2_S transformation, shuttle effect of soluble LiPSs, sluggish reaction kinetics, and formation of lithium dendrites [12,13]. Among them, the shuttle effect has a remarkable impact on the performance of LSBs, which can result in a decrease in LSBs’ cycling performance and a significant decrease in capacity [14,15].

**Scheme 1:**
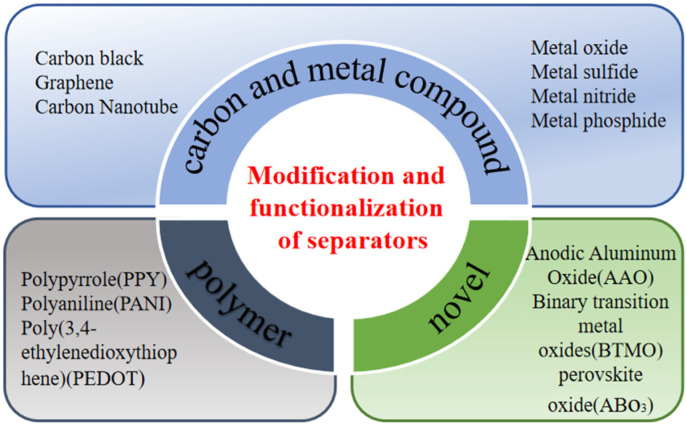
Various materials used for modification and functionalization of separators.

To address the previously stated issues, scientists have suggested various approaches such as designing cathode structures, employing novel sulfur-based host materials, optimizing electrolytes, and modifying and functionalizing separators, among others [16,17,18]. Among them, for the entire LSB device structure, although the separator, which avoids the direct electrical contact between the anode and cathode and also provides a channel for ion transport, is not an active component in the battery, it occupies a central place in determining the cost, cycle life, safety, and overall performance of the battery [19,20,21]. Consequently, the alteration and operationalization of the commercial separator serve as a potent tactic to enhance battery efficiency. Moreover, utilizing appropriate material systems to modify and functionalize the separator can not only restrain the shuttle effect but also accelerate electrochemical reaction kinetics, thereby improving the utilization rate of active materials [22]. This review comprehensively reviews the recent advances in functional separators for LSBs with various carbon and metal compound materials, polymer materials, and novel separators in recent years, as shown in Scheme 1. Detailed preparation processes, morphological and performance characterization as well as advantages and disadvantages are summarized, aiming at a fundamental understanding of the mechanism of battery performance improvement. Furthermore, the prospects for the development of advanced separators are discussed.

## 2. Modified and Functionalized Separators

In LSBs, the separator mainly blocks the transmembrane transport of LiPSs through adsorption, shielding, and screening, thereby suppressing shuttle effects and battery self-discharge phenomena [23,24]. In terms of suppressing lithium dendrites, improving the mechanical strength of the separator can avoid dendrite piercing. But a more effective method is to optimize the separator to improve the surface reaction current distribution of the lithium negative electrode and achieve uniform deposition of the lithium negative electrode [25]. In terms of improving the thermal safety of batteries, providing additional mechanical support to the separator can effectively reduce the thermal shrinkage rate of the separator [26,27,28]. Concurrently, creating innovative heat-resistant separators significantly enhances battery thermal safety. On the basis of analyzing the mechanism of separator action, this paper encapsulates the advancements in research on separators in LSBs and anticipates their forthcoming developments, in an effort to provide strong guidance for the development of LSBs.

### 2.1. Carbon and Metal Compound Materials

Carbon materials represent one of the oldest and most frequently employed materials for separator enhancement. They boast excellent electrical conductivity, a diverse array of functional groups, an adjustable porous structure, and a substantial surface area [29,30]. Coating carbon materials on the cathode side of the separator facilitates efficient electron transfer and enhances the utilization of cathode active materials [31]. Therefore, as a material with reasonable prices and convenient research methods, various carbon materials are favored by researchers, such as carbon black (Ketjen Black, super P), graphene, carbon nanotubes, etc.

**Figure 1:**
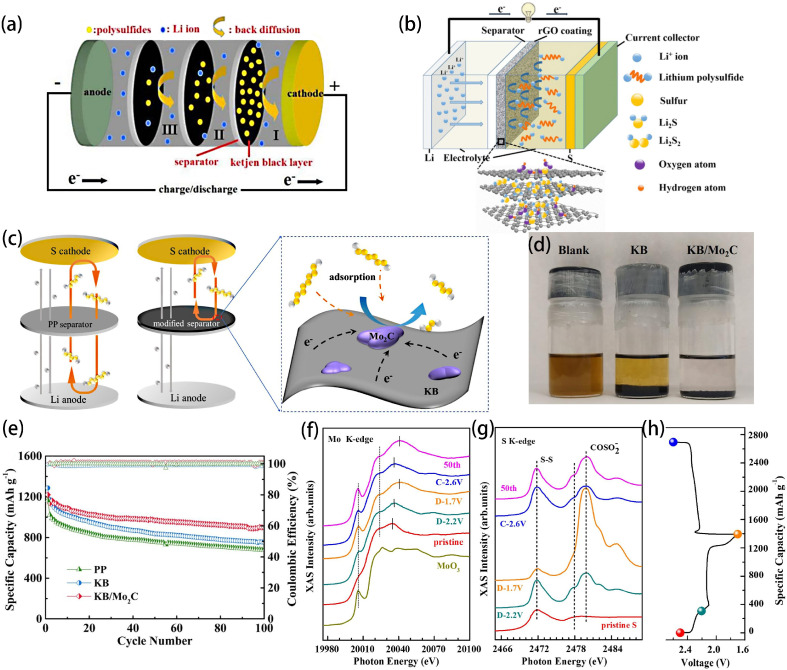
(**a**) The configuration of LSB with three layers of modified separators [32]. Copyright © 2017, with permission from John Wiley and Sons. (**b**) The schematic representation of a LSB incorporating a RGO-coated separator [35]. Copyright © 2015, with permission from IOP Publishing. (**c**) Schematic diagram of a LSB with KB/Mo_2_C-modified separator. (**d**) Photograph of the Li_2_S_6_ adsorption test: KB-modified and KB/Mo_2_C-modified separators. (**e**) Cycle performance at 0.2 C; ex situ XANES of (**f**) Mo K-edge and (**g**) S K-edge for the separator at certain states of charge/discharge during the first cycle and after 50 cycles. (**h**) The corresponding discharge–charge curves [39]. Copyright © 2021, with permission from Elsevier.

Among numerous carbon materials, Ketjen Black (KB) is a unique conductive carbon black with a high surface area and pore volume that facilitates the accommodation of polysulfides and provides additional positions for insoluble lithium sulfide. Tang et al. used modified separators coated with a thin KB conductive layer to suppress the shuttle effect in LSBs, as shown in Figure 1a [32]. They focused on studying how different layers of modified separators impact the performance of LSBs. Figure 1a illustrates the battery structure featuring a three-layer modified separator. The KB layer attached to the first modified membrane surface hampers polysulfide migration, whereas the KB layers on the subsequent two layers block the diffusion of polysulfides that escape from the first layer. When employing three modified separators, the battery demonstrated increased initial discharge capacity, credited to the improved electronic conductivity in the KB layer and the novel battery architecture. Graphene, a prototypical two-dimensional material, boasts exceptional mechanical flexibility, a substantial surface area, and high electrical conductivity, making it extensively employed in electrochemical energy storage devices. Graphene oxide (GO), as a derivative of graphene material, possesses good mechanical strength [33,34]. As early as 2015, Lin et al. prepared separators coated with a reduced graphene oxide (RGO) layer (Figure 1b). LSBs with RGO-coated separators exhibited better electrochemical performance [35]. The distinctive porous nature, high conductivity, and diverse functional groups of the RGO coating not only hindered polysulfide diffusion but also prominently expanded the conductive surface between the separator and the cathode. Carbon nanotubes are characterized by their superior electrical conductivity, mechanical strength, chemical stability, flexibility, and surface activity. The separator materials can effectively improve the performance and cycle life of LSBs [36]. Back in 2012, Professor Manthiram and colleagues suggested the creation of a freestanding multi-walled carbon nanotube (MWCNT) paper to serve as an interlayer in LSBs. The porous MWCNT paper is capable of absorbing polysulfides, thereby hindering their shuttling due to physical constraints [37].

The introduction of carbon materials as a separator coating inhibits lithium polysulfide shuttling and promotes polysulfide conversion. However, with the increase in sulfur surface density, the role of carbon material alone is far from satisfying the demand [38]. Therefore, combining carbon materials with other functional materials is a feasible strategy for achieving better performance in LSBs.

He et al. synthesized KB/Mo_2_C composites through the heat treatment of a KB and molybdenum precursor mixture. These composites were designed to serve as multifunctional decorative separators for LSBs, as shown in Figure 1c [39]. Mo_2_C particles were deposited on KB via carburizing, with Mo_2_C nanoparticles acting as adsorption and catalytic sites to capture LiPSs, thereby accelerating LiPS conversion kinetics and enhancing sulfur utilization. The KB/Mo_2_C-modified separators exhibited good mechanical stability. Through visual adsorption testing (Figure 1d), we can observe that the solution containing KB/Mo_2_C is almost colorless compared to the solution containing only KB and the pristine Li_2_S_6_ solution. The initial discharge capacity of the LSB equipped with a KB/Mo_2_C-modified separator reached 1221 mAh g^−1^ at 0.2 C. After 100 cycles, the capacity retention rate remained at 74%, significantly outperforming both the pp separator and the KB-modified pp separator, as shown in Figure 1e. X-ray absorption near-edge structure (XANES) (Figure 1f–h) further elucidated that the electron transfer between Mo_2_C and LiPS took place during the charging and discharging processes of Mo and LiPS. The strong affinity between Mo_2_C and LiPS resulted in the formation of stable C-Mo-S bonds on the Mo_2_C surface after several cycles. By employing a KB/Mo_2_C-modified separator featuring active LiPS capture sites, the shuttle effect can be significantly mitigated, leading to enhanced cycling efficiency of LSBs.

The aforementioned findings indicate that incorporating metal compound materials significantly improves both the conductivity and the mechanical steadiness of the separator. The incorporation of metal compounds can markedly improve the speed at which lithium ions are transferred within the separator, thereby boosting the battery’s charging and discharging efficiency and performance.

**Figure 2:**
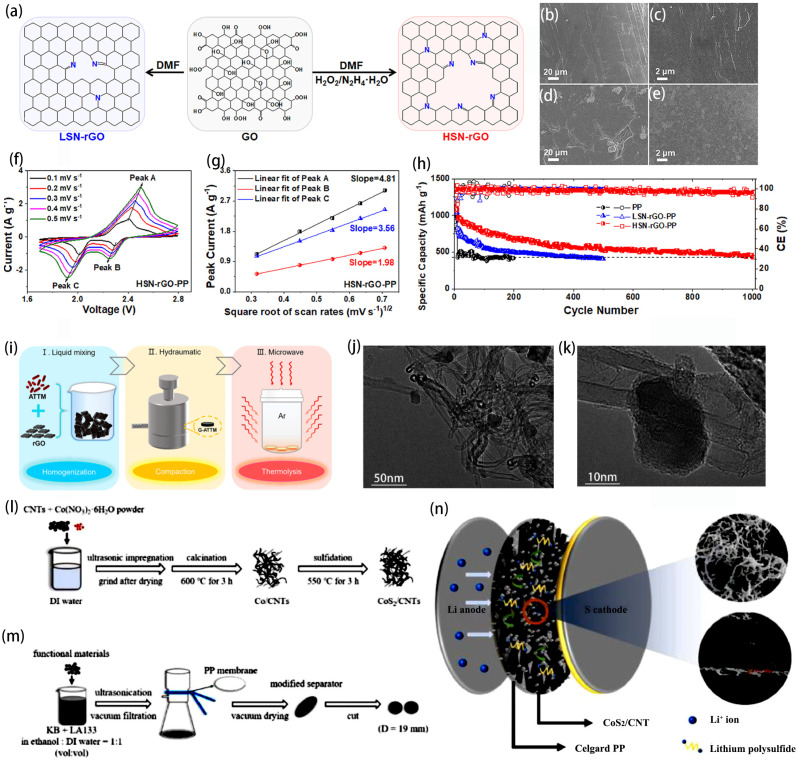
(**a**) Schematic illustration for the preparation process of HSN-rGO; SEM images of fresh Li (**b**,**c**) and Li metal anodes of the LSBs with HSN-rGO-PP (**d**,**e**) after 250 cycles at 1 C; (**f**) CV curves at different scan rate of the LSBs and (**g**) the corresponding linear fitting based on Randles–Sevick equation of the LSBs with HSN-rGO-PP; (**h**) long-term cyclic stability over 1000 cycles at 1 C of the LSBs with PP, LSN-rGO-PP, and HSN-rGO-PP, respectively [42]. Copyright © 2021, with permission from Elsevier. (**i**) Schematic diagram of mwGMSMO synthesis process [43]. Copyright © 2021, with permission from American Chemical Society. (**j**,**k**) TEM images of CoS_2_/CNT materials at different magnifications; (**l**) synthesis of CoS_2_/CNTs; (**m**) fabrication of a separator modified with functional materials; (**n**) application diagram of CoS_2_/CNT separator in LSBs [44]. Copyright © 2023, with permission from Elsevier.

Despite the excellent mechanical strength and flexibility of the aforementioned graphene, the interlayer space of pure graphene is relatively large, which can easily lead to the polarization failure of LSBs. By introducing metal compounds or heteroatoms, the structure of graphene can be adjusted to enhance its interlayer bonding and improve its chemical affinity with polysulfides, thereby enhancing the battery’s cycling performance [40,41]. Xiao et al. developed an innovative porous rGO characterized by a high specific surface area and high N doping (HSN-rGO) (Figure 2a) [42]. The high N doping content in HSN-rGO enhances its ability to absorb more LiPSs, and the abundant N sites in HSN-rGO significantly accelerate the redox reactions of LiPSs. HSN-rGO effectively mitigates the corrosion of LiPSs on the metal anode, preserving a smooth surface on the lithium metal negative electrode after cycling, which closely resembles that of fresh lithium metal (Figure 2b–e). HSN-rGO has a strong adsorption capacity for LiPS. The LSBs with HSN-rGO-PP exhibited the highest fitting slope which can be obtained by the Randles–Sevick equation (Figure 2f,g). Utilizing HSN-rGO-PP separators in LSBs led to quicker diffusion of Li^+^ and enhanced kinetic conversion of LiPSs. The initial discharge capacities of LSBs using HSN-rGO-PP separators were 1372.8 mAh g^−1^ and 681.5 mAh g^−1^ at 0.1 C and 2 C, respectively, and the capacity decaying rate was only 0.055% for 1000 cycles at 1 C (Figure 2h). Further, Zhao et al. synthesized multifunctional rGO/MoS_2_/MoO_2_ composites (mwGMSMO) by the microwave method for the coating layer of commercial polypropylene (PP) membranes (Figure 2i) [43]. RGO’s high conductivity coupled with the excellent catalytic and chemisorptive activities of MoS_2_/MoO_2_ facilitated the conversion of lithium polysulfide.

Similarly, composites of carbon nanotubes and metal compounds as Li-S battery separator materials can also enhance the electrochemical performance of the system. Zhu et al. synthesized polar CoS_2_ materials from Co(NO_3_)_2_ and CH_4_N_2_S and prepared the modified separator coated with CoS_2_/CNT by vacuum filtration and through a two-step pyrolysis method (Figure 2l,m) [44]. Figure 2n shows the schematic diagram of the CoS_2_/CNT separator used in a Li-S battery. The porous structure and high conductivity of CoS_2_/CNT promote the effective adsorption of LiPSs and enhance their catalytic conversion. CoS_2_’s polarity facilitates a chemical reaction with LiPSs, forming Co-S bonds, while the ordered carbon nanotubes create a robust conductive network. By adsorbing LiPSs on the separator’s positive side, further catalytic conversion is promoted, reducing the dissolution and diffusion of polysulfides. From Figure 2j,k, it can be seen that the CoS_2_ particles were successfully immobilized on the outer wall of CNTs, while the inner wall of the CNTs functioned as a conductive substrate, thereby accelerating electron conduction. The incorporation of inorganic metal sulfides into the modified CoS_2_/CNT layer boosted the use of reactive sulfur components by enhancing the chemisorption of LiPSs. LSBs using CoS_2_/CNT-PP separators exhibited good electrochemical performance.

Both graphene and CNTs have received widespread attention owing to their high electrical conductivity. Some scholars have found that their combination is beneficial to battery performance [45]. Gao et al. prepared graphene/CNT (G/CNT)-modified separators with high conductivity [46]. The conductivity of the G/CNT layer and the complex 3D structure had a synergistic effect in controlling the shuttling effect of polysulfides. In addition, the G/CNT layer can compensate for the effect of sulfur cathode morphology deterioration, which greatly improves the performance of LSBs. Utilizing GO and CNT simultaneously ensnares sulfur through physical confinement and chemical absorption while offering high porosity and enhanced conductivity for rapid ion and electron transfer [47]. Lee et al. developed a novel flexible carbon membrane sandwich consisting of graphene oxide, carbon nanotubes, and glass fiber (GF) by vacuum filtration method [48]. Hydrophilic functional factions within the GO layer bind lithium polysulfide that is dissolved in liquid electrolytes, whereas the CNT layer improves ionic conductivity. GO/CNT membranes’ superior physical and electrochemical characteristics substantially reduce the shuttling effect in LSBs.

Graphene and carbon nanotubes are the most promising candidates for improving the performance of LSBs, which can be applied not only to the cathode but also to the Li-S battery interlayer. Enhancing the battery’s electrochemical efficiency is achievable through the incorporation of heteroatoms or their amalgamation with metallic compounds, as we can clearly see from Table 1. In addition to carbon black, graphene, and carbon nanotubes, which are three commonly used carbon materials, materials like 3D porous carbon and graphitic carbon materials are often used to modify commercial separator efforts. It is becoming particularly important to explore more modified materials.

**Table 1:** A summary of electrochemical properties of coating separators of carbon materials for LSBs.

Separator	S Loading (mg/cm^2^)	Initial Capacity (mAh/g)/Rate	Cycle	Final Capacity (mAh/g)/Rate	Separator Coating Loading (mg/cm^2^)	Ref.
KB@PP	1.5	1430/0.1 C	150	770/2 C	-	[32]
RGO@PP	1.5	1067/0.2 C	100	878/0.2 C	-	[35]
MWCNT interlayer	-	1446/0.2 C	50	962/0.2 C	-	[37]
KB/Mo_2_C@PP	1.2	1221/0.2 C	100	898/0.2 C	0.6	[39]
HSN/rGO/@PP	-	957.1/1 C	1000	433.1/1 C	0.35	[42]
rGO/MoS_2_/MoO_2_/PP	1–1.2	1497/0.2 C	250	790/0.5 C	0.3	[43]
CoS_2_/CNTs@PP	-	1195.1/0.2 C	500	587.7/1 C	-	[44]
G/CNT@PP	1.7–2	1200/0.2 C	100	815/0.5 C	0.176	[46]
GO/CNT interlayer	1.1	1591.56/0.2 C	50	1000/0.2 C	-	[48]

The materials used for separators mentioned above, such as graphene, CNTs, and various other carbon materials, are preferred by earlier researchers for their lightweight nature, superior conductivity, and extensive specific surface area, enabling them to restrict polysulfides physically. Recently, scientists have proposed that metal compounds can create various adsorption sites to reduce polysulfides’ shuttling effect and hasten their redox conversion [49]. Transition metal compounds (TMCs) are highly beneficial as catalysts in sulfur redox reactions. Compounds containing polar oxygen possess solitary electron pairs on the oxygen atom, leading to increased binding energies between metal oxides and polysulfides [50,51]. Metal sulfides, metal selenides, metal nitrides, metal phosphides, and metal carbides can be effective catalysts for the conversion of polysulfides. However, most metal compounds have poor electrical conductivity. Combination with conductive porous carbon materials has become a widely accepted approach [47,52]. Such composites exhibit strong chemisorption of sulfides and can better inhibit the shuttle effect, as shown in Table 2.

**Figure 3:**
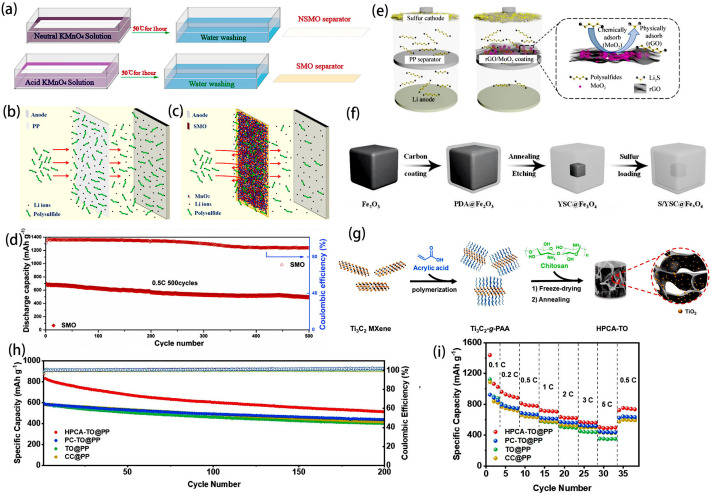
(**a**) Schematic diagram of manufacturing methods for NSMO and SMO separators. Schematic diagram of prohibited shuttle by the (**b**) PP and (**c**) SMO separators. (**d**) Long cycle performance of LSBs with SMO separators after 500 cycles at 0.5 C [57]. Copyright © 2021, with permission from Elsevier. (**e**) Mechanism diagram of rGO/MoO_2_-modified PP separator [58]. Copyright © 2021, with permission from Springer Nature. (**f**) Schematic diagram of the synthesis process of the S/YSC@Fe_3_O_4_ [59]. Copyright © 2017, with permission from John Wiley and Sons. (**g**) Schematic diagram of HPCA-TO. (**h**) Cycle performance and corresponding Coulombic efficiency of batteries with different separators at 1 C. (**i**) Rate capacity of the batteries with the different separators at different current rates [60]. Copyright © 2022, with permission from Elsevier.

Compared to non-polar carbon-based materials, polar metal oxides demonstrate stronger chemical interactions with polysulfides and greater catalytic efficiency. The polar bonding between the metal cation and oxygen anion creates sufficient polar active centers to effectively anchor polysulfides, and thus a large number of inexpensive metal oxide materials are widely used in Li-S battery separators [53,54,55]. Ban proposed a functional separator modified by MoO_2_ nanosheets (MONS) [56]. The potent adsorption and catalytic properties of MoO_2_ nanosheets significantly enhance the redox kinetics of polysulfides, thereby inhibiting the shuttle effect. Wang et al. employed the oxidation properties of acidic KMnO_4_ solution to treat PP separators, immersing them in the solution for 1 h, as shown in Figure 3a [57]. An ultrathin self-assembled MnO_2_ layer was directly applied to one side of the separator, referred to as SMO. In contrast, the separator prepared with a neutral KMnO_4_ solution was named the NSMO separator. The self-assembled MnO_2_ layer exhibits a combined impact of adsorption and catalytic transformation on polysulfides, thus efficiently inhibiting the shuttle effect. Figure 3b and Figure 3c show the schematic diagrams of PP and SMO separators prohibiting the shuttle, respectively. Dissolved polysulfides can migrate through the PP separator to the anode, causing shuttle effects that disrupt cycle stability and reduce active material utilization. The self-assembly of a polar manganese dioxide layer via this simple chemical process largely prevents the shuttle phenomenon and accelerates reuse through chemical adsorption and catalytic promotion. Consequently, batteries employing SMO separators exhibit significantly improved cycle stability compared to those using PP separators. The capacity decaying value (per cycle) of the battery with SMO separators is only 0.058% after 500 cycles at 0.5 C (Figure 3d). Xu et al. synthesized rGO/MoO_2_ composites and applied them to modifying pp separators [58]. From Figure 3e, it can be observed that rGO functioned as a conductive physical barrier to impede polysulfide ion shuttling, while acting as a matrix for uniformly dispersing MoO_2_. The uniformly dispersed polar MoO_2_ on rGO offers numerous chemical adsorption and catalytic sites, accelerating polysulfide conversion and preventing shuttle effects. The electrochemical performance of batteries assembled with rGO/MoO_2_-modified separators was significantly improved due to the synergy of physical adsorption, chemical interaction, and catalysis in rGO/MoO_2_ composites.

Due to the poor conductivity of most polar compounds, it is not conducive to rapid electron transport, which affects the multiple properties. He et al. designed the yolk–shell carbon@Fe_3_O_4_ (YSC@Fe_3_O_4_) nanobox structure to serve as highly effective sulfur hosts for LSBs (Figure 3f) [59]. The carbon shells act as nanoscale electrochemical reaction chambers and physically limit the diffusion of polysulfides. Polar Fe_3_O_4_ cores serve as centers for polysulfide capture by deeply chemically interacting with the polysulfides, which are predominantly anchored and kept inside the nanobox. Due to the presence of a highly conductive Fe_3_O_4_ core combined with a carbon shell, electron transport is accelerated, aiding in the battery’s electrochemical reaction kinetics. Shi et al. prepared hierarchical porous carbon aerogels (HPCA-TO) embedded with small TiO_2_ nanoparticles. The preparation schematic diagram of HPCA-TO is shown in Figure 3g. They used polyacrylic acid-grafted Ti_3_C_2_ MXene as a two-dimensional template and TiO_2_ source, while chitosan served as the carbon precursor [60]. The HPCA-TO was filtered onto commercial pp separators, serving as a functional intermediate layer for the sulfur cathode. HPCA-TO is equipped with small-sized polar TiO_2_ nanoparticles, aiding in the chemisorption of dissolvable polysulfides, and its two-dimensional carbon framework enhances electrode conductivity, complemented by a hierarchical porous structure that promotes ion diffusion. As a result, HPCA-TO effectively inhibited the polysulfide shuttle effect and significantly enhanced the kinetics of their conversion. Consequently, the LSBs with modified separators demonstrated superior rate capabilities and cycle performance (Figure 3h,i).

Metal sulfides possess superior electrical conductivity and optical properties than metal oxides. MoS_2_, as one of the metal sulfide materials, is widely used in energy storage and photocatalysis. The substance demonstrates superior properties in diffusing lithium ions. Notably, MoS_2_ spheres are characterized by numerous active sites and rich interfaces, which are conducive to enhancing the kinetics of redox reactions and catalytic processes [61,62]. Liu et al. prepared MoS_2_-coated separators that acted as effective barriers to inhibit the shuttle effect in LSBs [63]. From the SEM images, it is evident that the MoS_2_-coated separator displayed a more uniform porous structure compared to the original separator. The MoS_2_-coated separator showcased initial specific capacities of 1308 mAh g^−1^ at 0.1 C and 1246 mAh g^−1^ at 0.2 C, outperforming the pristine separators. Wang et al. prepared a C@MoS_2_-modified separator [64]. In contrast to other modified separators, the C@MoS_2_ separator demonstrated an effective effect in converting polysulfide. The LSB with a C@MoS_2_ separator had an initial specific capacity of up to 1109 mAh g^−1^ at 0.2 C.

**Figure 4:**
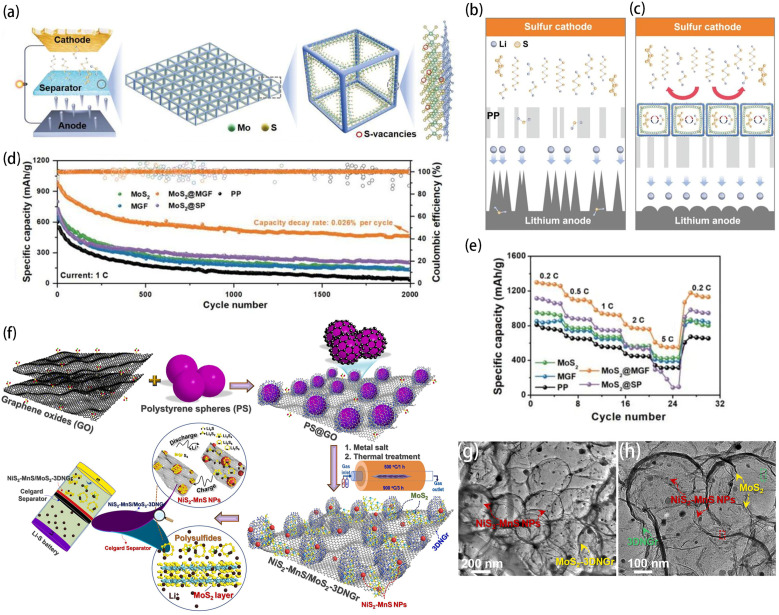
(**a**) Schematic of MoS_2_@MGF as an interlayer for LSBs. Schematic of LSBs with PP (**b**) and MoS_2_@MGF/PP (**c**) separators; (**e**) rate capabilities; and cycling performance (**d**) at 1 C for LSBs with MoS_2_/PP, MGF/PP, MoS_2_@MGF/PP, MoS_2_@SP/PP, and PP [67]. Copyright © 2024, with permission from John Wiley and Sons. (**f**) Schematic diagram of the synthesis process of the NiS_2_-MnS/MoS_2_-3DNGr hybrid. (**g**,**h**) TEM images of the NiS_2_-MnS/MoS_2_-3DNGr hybrid [70]. Copyright © 2022, with permission from Elsevier.

Other types of MoS_2_-based coating materials, such as MoS_2_/activated carbon and MoS_2_/graphene, often contain highly graphitized carbonaceous materials. The highly disordered porous structure inherent in these coatings is not well suited for regulating Li^+^ flux [65,66]. Gao et al. have developed a new type of 2D mesoporous graphitic framework (MGF) consisting of interconnected cubic cages, derived from self-assembled nanocrystal superlattices as the supporting skeleton for the epitaxial growth of MoS_2_ with abundant sulfur vacancies (S-vacancies), as shown in Figure 4a [67]. The MoS_2_@MGF membrane is used as a multifunctional interlayer for LSBs to improve the overall battery performance. The exceptional electrical conductivity and ordered mesoporous structure of the MGF can promote the uniform distribution and high-flux diffusion of Li^+^, resulting in dendrite-free lithium anodes and shuttling-free sulfur cathodes. MoS_2_@MGF possesses numerous curvature-induced strains and abundant S-vacancies in the encapsulated MoS_2_, which can uniformly and smoothly trap polysulfides in its cubic cage, thus facilitating the subsequent adsorption–diffusion conversion process (Figure 4b,c). The resulting 2D MoS_2_@MGF heterostructure exhibits improved polysulfide adsorption capacity, enhanced catalytic activity in LiPS conversion, and improved sulfur utilization. The MoS_2_@MGF/PP battery exhibits shuttle-free and dendrite-free behavior, resulting in enhanced rate capability (Figure 4e) and exceptional long-term cycling stability (Figure 4d). In addition, bimetallic sulfides have been shown to be highly effective electrocatalysts for the redox reactions of polysulfides due to the synergistic effect of their multiple catalytic activity centers and their components [68,69]. Doan’s team has developed a highly efficient and multifunctional separator for LSBs. This coating material consists of binary NiS_2_-MnS nanoparticles dispersed on ultrathin MoS_2_ nanosheets and supported by an N-doped three-dimensionally interconnected hollow graphene sphere (3DNGr) substrate (referred to as NiS_2_-MnS/MoS_2_-3DNGr) [70]. Figure 4f shows the synthesis process of hierarchical NiS_2_-MnS/MoS_2_-3DNGr heterostructure hybrid materials. The hierarchical heterogeneous structure of the material can be clearly seen by TEM images (Figure 4g,h). The MoS_2_ nanosheets demonstrate a high adsorption capacity for polysulfides, while the uniformly distributed NiS_2_-MnS nanoparticles act as additional adsorption sites for polysulfide intermediates and offer catalytically active sites that enhance the redox reactions of these intermediates. LSBs equipped with NiS_2_-MnS/MoS_2_-3DNGr-modified separators demonstrate superior electrochemical performance compared to those using Celgard separators. The initial capacity of the battery reached 1011 mAh g^−^^1^ at 0.1 C, and the capacity faded by merely 0.12% per cycle over a duration of 200 cycles.

Metal nitrides with polar properties are generally highly conductive and have abundant sites to trap and convert polysulfides, which can suppress the shuttle effect of LSBs. Fan et al. obtained 2D TiN@C sheets through in situ nitriding of MXene. These sheets were then combined with graphene to create a separator coating for LSBs [71]. Excellent conductivity TiN@C not only avoids the deposition of Li_2_S_2_/Li_2_S but also provides numerous active sites, which is beneficial for capturing and converting dissolved LiPSs. Additionally, the incorporation of graphene effectively hinders the shuttle effect of LiPS. Based on the above advantages, LSBs with TiN@C/G-coated separators demonstrate outstanding electrochemical performance, achieving an initial capacity of up to 1490.2 mAh g^−1^ at 0.1 C.

Compared with conductive metal nitrides, metal phosphides have the advantage of simpler synthesis. Metal phosphides, especially transition metal phosphides, have excellent thermal stability and excellent electrocatalytic performance, making them suitable for LSBs. Metal phosphides, as polar compounds, can endow composite materials with excellent adsorption capacity [72,73]. When using metal phosphide alone to modify separators, metal phosphide cannot provide sufficiently satisfactory conductivity to facilitate charge transfer. Liu et al. designed and grew Ni_12_P_5_ nanoparticles on a reduced graphene oxide (Ni_12_P_5_@rGO) framework and used it as a modifier for separators in LSBs [74]. The synergistic interaction between Ni_12_P_5_ particles with high binding energy and reduced graphene effectively mitigates the shuttle effect, enhancing battery lifespan. Li-S battery with Ni_12_P_5_/rGO-modified separators had a capacity of 632 mAh g^−1^ after 500 cycles at 1 C. Wu et al. combined the carbon skeleton with polar Co_2_P through a one-step in situ growth method. Co_2_P with a carbon skeleton layer can effectively adsorb long-chain LiPS and inhibit the shuttle effect to a large extent [75]. Although the preparation of metal phosphides is simple and mild, the synthesis of metal phosphides requires a phosphorylation process, which can produce some toxic gases. Moreover, they are easily oxidized under environmental conditions. Therefore, there is an urgent need to develop a series of phosphides with excellent properties.

In summary, we can see the advantages of carbon materials as LSB separator materials from Table 1. However, carbon materials alone may not be able to effectively capture and immobilize polysulfides generated by the lithium–sulfur reaction. The various metal compound separators mentioned above can effectively inhibit the shuttling effect of polysulfides in LSBs and reduce the loss of active materials; however, the conductivity of many metal compounds is relatively low, which may affect ionic conduction. Based on the characteristics of the two materials, the excellent electrical conductivity of carbon materials and the chemisorption ability of metal compounds complement each other, thereby improving the overall performance of the separator. By adjusting the ratio and structure of carbon materials and metal compounds, polysulfides can be effectively controlled, and conductivity can be optimized. However, some metal compound materials have a high density, which may increase the overall weight and volume of the battery, thus affecting the energy density of the LSB. Although the conductivity of some metal compounds can be improved by modification, there are still some metal compounds that are not as conductive as polymer materials or other materials, limiting their applications. In future development, combinations of novel carbon materials (such as nanocarbon, functionalized graphene, etc.) and innovative metal compounds can be explored to improve the overall performance of the separator.

**Table 2:** A summary of electrochemical properties of coating separators of metal compound materials for LSBs.

Separator	S Loading (mg/cm^2^)	Initial Capacity (mAh/g)/Rate	Cycle	Final Capacity (mAh/g)/Rate	Separator Coating Loading (mg/cm^2^)	Ref
PP/MONs	1.9	1246/0.05 C	200	908/0.1 C	-	[56]
PP/SMO	1–1.2	733/0.5 C	150	603/0.5 C	0.02	[57]
PP/rGO/MoO_2_	1.1	1290.5/0.2 C	200	757.5/0.2 C	0.17	[58]
PP/HPCA-TO	0.8	725/1 C	200	513/1 C	0.4	[60]
Celgard/MoS_2_	-	1308/0.1 C	500	796/1 C	-	[63]
PP/C/MoS_2_	1.3	1109/0.2 C	200	1002/0.5 C	0.13	[64]
PP/MoS_2_@MGF	1	1300/0.2 C	2000	463/1 C	-	[67]
Celgard/NiS_2_-MnS/MoS_2_-3DNGr	1	1011/0.1 C	200	302.2/3 C	2.16	[70]
Celgard/TiN@C/G	0.71	1490.2/0.1 C	600	635.8/1 C	0.2	[71]
PP/Ni_12_P_5_/rGO	1.1–1.5	1428/0.2 C	100	899/0.5 C	0.4	[74]
Celgard/Co_2_P/Co/C	2.68	1310/0.5 C	100	800/0.5 C	-	[75]

### 2.2. Polymers

Polymer materials can be used in the field of separator modification for LSBs because they have different functions and endow the separator with different properties. Polymer materials are thin, lightweight, and electrically conductive, and their surfaces are usually distributed with micropores, mesopores, or multilayered pores, which can inhibit polysulfides shuttling by the principle of pore size sieving [76,77]. In addition, there are various types of functional groups in polymers that can inhibit polysulfide shuttling through electrostatic repulsion and chemisorption. Various conductive polymers, including polypyrrole (PPy), polythiophene (PEDOT), polyaniline (PANI), and others, serve as separators or layers in LSBs, owing to their porous structure and superior flexibility [78,79]. The molecular chains of these polymers are typically rich in polar functional groups, which can help prevent the loss of lithium polysulfide [80].

#### 2.2.1. PPy

Conducting PPy possesses high electrical conductivity, good chemical stability, and excellent electrochemical redox reversibility. Due to its excellent adsorption capacity for LiPSs and high electrolyte affinity, PPy is widely used in optimizing the performance of separators and preventing the shuttling of LiPSs [81,82].

**Figure 5:**
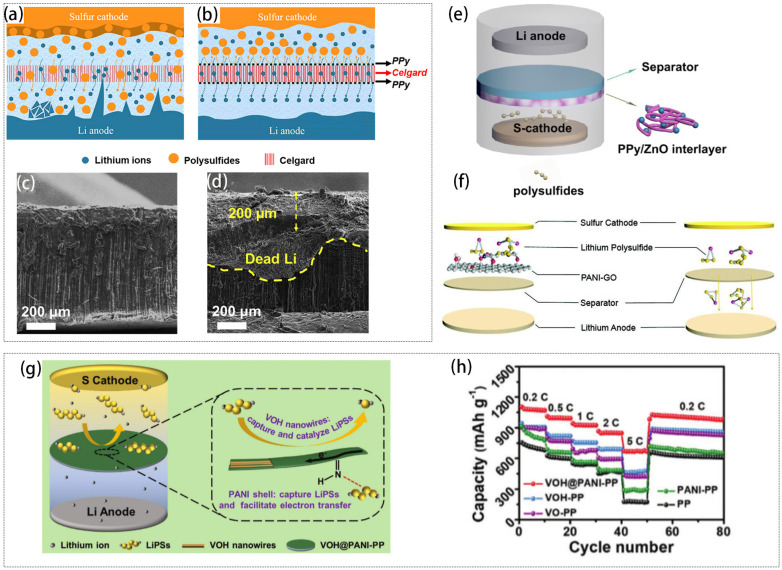
Schematic illustration of the LSBs with (**a**) a regular Celgard separator and (**b**) a PPy-modified separator; cross-section SEM images of Li metal anode of the LSBs with (**c**) PPy-modified separators and (**d**) regular Celgard separators after 250 cycles at 0.5 C [85]. Copyright © 2019, with permission from Elsevier. (**e**) A schematic of the cell with PPy/ZnO interlayer [86]. Copyright © 2018, with permission from Springer Nature. (**f**) Schematic diagram of a PANI-GO interlayer inserted between cathode and separator in LSBs [90]. Copyright © 2018, with permission from Royal Society of Chemistry. (**g**) Schematic illustrating the mechanism of the high-performance battery equipped with VOH@PANI-PP separator. (**h**) Rate performance of the battery equipped with the VOH@PANI-PP separator at different current densities [92]. Copyright © 2021, with permission from John Wiley and Sons.

Ma et al. modified Li-S battery separators using PPy nanowires, PPy nanotubes, and rGO as early as 2015 to observe the effects of different modified materials [83]. It was found that PPy adsorbed lithium polysulfide more strongly than rGO and the PPy-modified separators had better wettability to the electrolyte, which enhanced the electrochemical performance of LSBs. Furthermore, Ma et al. self-assembled PPy nanotubes into PPy nanotube film (PNTF), which served as a functional interlayer. The high BET-specific surface area of the PPy nanotubes enabled the PNTF to efficiently inhibit polysulfide solubilization and migration, effectively suppressing the shuttle effect during the charging and discharging processes [84]. Li et al. designed a stable cathode–separator–anode interface by introducing an ultrathin and lightweight PPy-functionalized layer on the surface of the Celgard separator through a simple gas-phase polymerization method (the working mechanism was shown in Figure 5a,b) [85]. The hydrophilic surface of PPy enhances the wettability of the electrolyte, resulting in a uniform flux of lithium ions and consistent deposition and stripping of lithium metal on the anode side. The PPy-modified separator provides a smoother lithium-metal anode surface than conventional separators (as shown in Figure 5c compared to Figure 5d) and maintains its structural integrity even after 250 charge/discharge cycles. LSBs with PPy-modified separators have a higher specific capacity (0.1 C, 1271 mAh g^−1^). The introduced PPy functionalized layer of the Celgard separator ensures the uniform flow of lithium ions on the one hand, thus realizing the uniform deposition and detachment of lithium metal on the lithium anode. On the other hand, it can effectively anchor lithium polysulfide and inhibit the lithium polysulfide shuttle effect. Additionally, the introduced PPy layer, with a weight of approximately 0.13 mg/cm^2^ and a thickness of about 60 nm, has minimal influence on the battery’s overall energy density.

In addition, PPy is able to adsorb polysulfides through H-bonding, while polar metal oxides can form chemical bonds with polysulfides to minimize the loss of active materials. Yin et al. developed a novel interlayer incorporating PPy and ZnO nanoparticles [86]. The battery structure featuring a PPy/ZnO intermediate layer is depicted in Figure 5e. The PPy/ZnO composite is uniformly coated on the membrane, forming a polysulfide-trapping interlayer. The combination of PPy and ZnO enhances the ability of the interlayer to capture polysulfides and avoids the poor electrical conductivity of pure ZnO interlayer. The prepared novel interlayer minimizes the shuttling of polysulfides, effectively protects the lithium anode, and improves the rate performance. In addition to combining PPy with metal compounds, Zhou et al. embedded conductive PPy into a two-dimensional ultrathin graphene layer (PPy/G) to prepare a multifunctional interlayer, which possesses a high concentration of unique pyrrole nitrogen and excellent wettability to the electrolyte [87]. PPy/G exhibits strong chemical interactions with polysulfides (PSs) and promotes the PSs redox reaction.

#### 2.2.2. PANI

PANI is a popular conductive polymer, which is widely used due to its excellent conductivity, porous properties, and good chemical stability. Its significant role in LSBs is attributed to its robust affinity towards lithium polysulfide [88,89]. Nonetheless, using general PANI solely as an intermediate layer material poses challenges, necessitating a self-supporting substance as a base for polymerizing aniline. Yin et al. synthesized a conductive PANI layer on the surface of a GO film using in situ chemical oxidative polymerization [90]. The schematic diagram of the polyaniline graphene oxide (PANI-GO) interlayer inserted between the sulfur cathode and separator of the Li-S battery is shown in Figure 5f. The PANI-GO interlayer effectively intercepted dissolved lithium polysulfides through a combination of physical barriers and chemisorption. Lithium–sulfur batteries incorporating this interlayer exhibited excellent cycling stability and superior rate performance.

**Figure 6:**
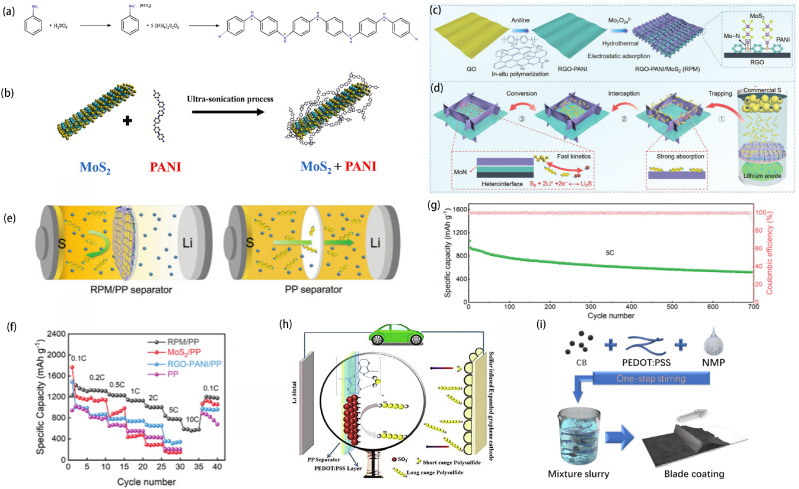
Polymerization scheme of aniline emeraldine salt chains (**a**); schematic illustration of the MoS_2_/PANi assembly (**b**) [93]. Copyright © 2021, with permission from Elsevier. (**c**) Schematic illustration of the fabrication of RPM and (**d**) RPM-modified separator for the inhibition and reutilization of LiPSs. (**e**) Schematic diagram of LiPSs trapping–interception–conversion process on RPM/PP (left) and severe shuttle effect on PP separator (right). (**f**) Rate performance of the cell with different separators. (**g**) Cycling performance of the cell with RPM/PP separator at 5 C [94]. Copyright © 2022, with permission from John Wiley and Sons. (**h**) Theoretical mechanism of the battery incorporating a PEDOT:PSS-coated separator and a sulfur-infused expanded graphene cathode [100]. Copyright © 2016, with permission from Royal Society of Chemistry. (**i**) Preparation of CB/PEDOT:PSS-modified separator [101]. Copyright © 2019, with permission from John Wiley and Sons.

In addition, some scholars have found that the combination of PANI with metal compounds can mitigate the shuttle effect. The transition metal oxide vanadium pentoxide (V_2_O_5_) is often used to modify the separator of LSBs and its strong chemical interaction with lithium polysulfide can greatly inhibit the shuttle effect [91]. The structural defects resulting from the long-range disordered structure of amorphous V_2_O_5_ (hydrated vanadium pentoxide, V_2_O_5_-nH_2_O, abbreviated as VOH) further enhance the catalytic activity for the conversion of lithium polysulfides. Chen et al. synthesized PANI-encapsulated amorphous vanadium pentoxide nanowires via in situ chemical oxidative polymerization and utilized them as key components to develop functional interlayers on commercial polypropylene separators [92]. The schematic diagram of a high-performance LSB equipped with a VOH@PANI-PP separator is shown in Figure 5g. Within the interlayer, the long-range disordered structure of amorphous V_2_O_5_ provides higher adsorption capacity compared to its crystalline form. The encapsulation of PANI enhances the conversion kinetics of LiPSs, accelerates electron transfer, and facilitates the reuse of inactive LiPSs in the electrolyte. Additionally, the imine (-N=) functional group in PANI improves its LiPS capture ability. These benefits collectively reduce shuttle effects during battery charging and discharging. The battery with the VOH@PANI-PP separator exhibits a high initial capacity and superior rate performance compared to other separators (Figure 5h). Daniele Versaci’s team synthesized PANI/MoS_2_ composites (Figure 6a,b) using a direct ex-situ method. They then coated a second layer, containing different amounts of MoS_2_ and PANI, onto the surface of the sulfur cathode [93]. MoS_2_ catalyzes the conversion reaction of lithium polysulfides, while PANI acts as a physical barrier to inhibit the migration of these polysulfides. This bilayer composite significantly enhanced the performance of the sulfur positive electrode.

Shi et al. proposed a Mott-Schott RGO-PANI/MoS_2_ (RPM) heterostructure-modified separator by vertically growing MoS_2_ arrays on PANI in situ reduced RGO (Figure 6c,d) [94]. The RPM demonstrates a “trapping-interception-conversion” mechanism for lithium polysulfides (Figure 6e). The unique “reservoir” structure combining MoS_2_ arrays and RGO-PANI substrate effectively utilizes the strong adsorption capacity of edge-rich MoS_2_, intercepting LiPSs efficiently. This modified layer features abundant edge sites, in situ heterogeneous interfaces, and efficient sites for polysulfide storage and conversion. Through the vertically aligned structure, MoS_2_ can effectively capture and convert polysulfides, while RGO-PANI provides good electron transport performance. The rate performance of the battery with RPM/PP is much higher than that of other interlayer materials (Figure 6f). Despite a high current density of 5 C, the battery equipped with an RPM/PP separator demonstrates a coulombic efficiency rate of 99.7% over 700 cycles (Figure 6g). This modified layer effectively inhibits the polysulfide shuttle effect and enhances sulfur utilization.

In addition, the performance of LSBs has been improved by combining PANI with some bimetallic oxides as separator materials. PANI itself is a conductive polymer, and combining it with bimetallic oxides significantly improves the electrical conductivity of the composite material, which in turn promotes the rapid conduction of electrons and improves the charging and discharging performance of the batteries [95].

Considering the large advantages of Prussian blue analogs (PBAs) as functional materials for separator modification, Jo’s team synthesized open-framework hollow Co–Fe Prussian blue analogs (CFPs) by oriented attachment (OA) [95]. They developed a hollow CFP encapsulated in polyaniline (CFP@PANI) and applied the synthesized CFP@PANI onto pp separators. Bimetallic CFP is characterized by its large specific surface area and potent catalytic function, with its PANI layer significantly contributing to the prevention of polysulfide dissolution, enhancing both conductivity and structural integrity. Li et al. synthesized polyaniline-coated cobalt–iron oxide (CFOP) composites by direct calcination and in situ oxidative polymerization [96]. The chemisorption of polysulphides by Co and Fe bimetallic oxides, as well as the enhancement of the electrical conductivity of the surface-covered polyaniline, effectively improved the cathode reaction kinetics. Subsequently, the integration of the CFOP separator with the S/KB cathode significantly improved the electrochemical performance of the battery. The specific capacity of the cell was as high as 1489.4 mAh g^−^^1^ at 0.1 C, and the cell was able to exhibit good cycling stability even at a higher sulfur content and higher current density. The combination of bimetallic oxides and PANI offers many advantages, especially in terms of battery performance, but a number of challenges still need to be addressed during fabrication and application in order to realize its widespread use in LSBs.

#### 2.2.3. PEDOT

In addition, the Bayer company (Germany) first synthesized poly(3,4-ethylenedioxythiophene) (PEDOT), a derivative of polythiophene (PTh), in 1988 [97]. As one of the derivatives of PTh, PEDOT is widely used in research fields such as batteries, OLEDs, and so on, due to its high electrical conductivity and excellent environmental stability. Nonetheless, the non-soluble characteristics of PEDOT restrict its use across various fields. As a water-soluble electrolyte, Polystyrene sulfonic acid (PSS), incorporating PSS into PEDOT to create a PSS-wrapped PEDOT structure markedly enhances its solubility in the solvent. SO_3_^−^ groups with a negative charge in PSS, PEDOT:PSS film, offer electrostatic protection against soluble lithium polysulfide via coulomb repulsion [98,99]. Therefore, it is also used as a cathode intermediate layer. Abbas et al. developed a bifunctional separator by spraying PEDOT:PSS onto the separator, as shown in Figure 6h [100]. In PSS, the negatively charged SO_3_− groups act as an electrostatic barrier, repelling soluble lithium polysulfides through Coulombic forces. Meanwhile, PEDOT promotes a chemical interaction with insoluble polysulfides such as Li_2_S and Li_2_S_2_. The distinctive bifunctional PEDOT:PSS separator design significantly reduces lithium polysulfide penetration, thereby greatly improving the suppression of the soluble lithium polysulfide shuttle and enhancing the cycle life of the LSB compared to the pristine separator. Yi et al. designed a bifunctional CB/PEDOT:PSS-modified separator to serve as a secondary current collector, significantly improving sulfur utilization (Figure 6i) [101]. The incorporation of PEDOT:PSS effectively inhibited polysulfide diffusion and facilitated lithium ion migration. The battery demonstrated an initial capacity of 1315 mAh g^−1^ at 0.2 C and retained a capacity of 956 mAh g^−1^ after 100 cycles.

In summary, we can see the advantages of polymers as LSB membrane materials. From Table 3, it can be seen that by combining with conductive materials such as carbon materials and metal compounds, the conductivity of the composite separator can be significantly improved, thereby enhancing the charging and discharging performance of the battery. The conductive polymers PPy, PANI, PEDOT, and conductive nanomaterials such as G and GO mentioned earlier have improved the electronic and ionic conductivity of the membrane. By compounding with metal compounds, the barrier effect of the membrane on polysulfides is enhanced, reducing the shuttle effect. And the modified polymer membrane has higher thermal stability, reducing the risk of material decomposition at high temperatures. With the rapid growth of the electric vehicle market, high-performance polymer separator materials will play a key role in improving the energy density and cycle life of lithium–sulfur batteries. In terms of future development, multilayer composite technology will be used to further enhance the comprehensive performance of the separator, achieve higher energy density and cycle life, and meet the higher performance requirements of LSBs.

**Table 3:** A summary of electrochemical properties of coating separators of polymer materials for LSBs.

Separator	S Loading (mg/cm^2^)	Initial Capacity (mAh/g)/Rate	Cycle	Final Capacity (mAh/g)/Rate	Separator Coating Loading (mg/cm^2^)	Ref.
PPy nanotube@PP	2.5–3	1200/0.2 C	200	865.4/0.2 C	1	[83]
PPy nanotubes film interlayer	2.5–3	1102/0.5 C	300	712/0.5 C	1	[84]
PPy/Celgard	1.2	985/0.5 C	250	805/0.5 C	0.13	[85]
PPy/ZnO interlayer	1.3	951/0.2 C	100	579/0.2 C	-	[86]
PPy/G@PP	1.0–1.5	1360/0.2 C	200	900/0.2 C	0.35	[87]
PANI-GO interlayer	1.96	1261/0.5 C	150	896/0.5 C	2.48	[90]
VOH@PANI-PP	1.2	1132.4/0.2 C	100	999.6/0.2 C	-	[92]
RGO-PANI-MoS_2_/PP	1	1233/0.5 C	200	827/0.5 C	0.24	[94]
CFP@PANI-PP	0.8–1	790.6/1 C	100	603.3/1 C	0.2	[95]
PP-CFOP	1.2	1489.4/0.1 C	100	818.5/0.1 C	0.15–0.20	[96]
PEDOT:PSS@PP/PE	0.9–1.1	901/0.5 C	500	596/0.5 C	-	[100]
CB/PEDOT:PSS@PP	1.6	1315/0.2 C	100	956/0.2 C	0.604	[101]

### 2.3. Novel Separators

With the wide application of LSBs in high-energy-density energy storage, research on novel separator materials has become increasingly important. As technology develops, researchers are continuously creating various types of innovative separator materials. These materials play a key role in enhancing battery performance by effectively inhibiting the migration of lithium polysulfide, thereby improving cycling stability and overall efficiency. The redesigned novel separators feature a variety of structures and properties that overcome the limitations of traditional separators.

**Figure 7:**
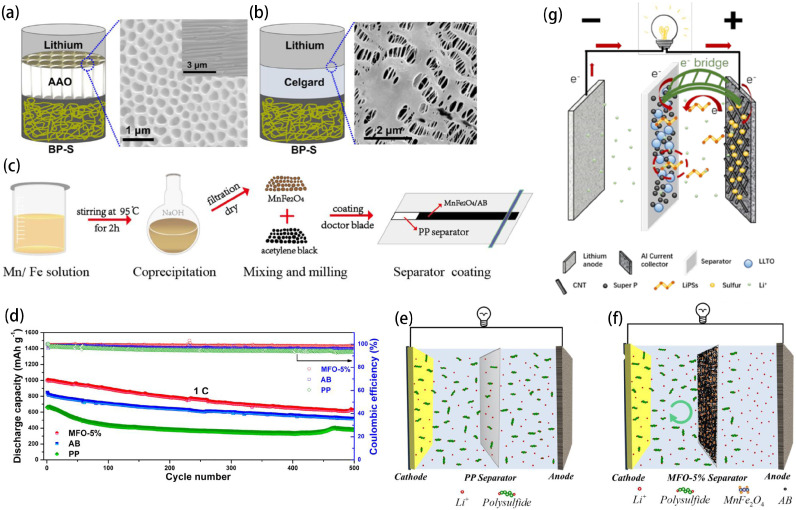
(**a**) The structure of LSB with AAO separator and SEM image of AAO surface. (**b**) The structure of a LSB with a Celgard separator and SEM image of the surface of the Celgard 2400 [103]. Copyright © 2020, with permission from American Chemical Society. (**c**) Schematic illustration of the fabrication process of MnFe_2_O_4_ and modifying separator. (**d**) The cycling performance of the batteries equipped with PP, AB, and MFO-5% separator after 500 cycles at 1 C. (**e**) The schematic of LSB configured with PP separator. (**f**) Schematic diagram of LSB with MFO-5%-modified separator configuration [105]. Copyright © 2020, with permission from Elsevier. (**g**) Schematic diagram for LLTO@Li-S battery [107]. Copyright © 2022, with permission from American Chemical Society.

Anodic Aluminum Oxide (AAO) membranes possess a nanoporous structure on the surface. AAO membranes have good thermal stability, chemical stability, and unique structure, and are widely used in optical devices, photocatalysis, energy, and other fields [102]. From the perspective of safety and cycle stability, Wang et al. first used a rigid and non-flammable AAO membrane as a separator for LSBs, as shown in Figure 7a,b [103]. This innovative separator features a unique combination of density and porosity, creating pathways that enable the rapid transport of lithium ions in the electrolyte. This reduces the generation of lithium dendrites and minimizes polysulfide shuttling. The LSB assembled with the AAO separator demonstrates a specific discharge capacity of 1334.7 mAh g^−1^ at 0.1 C and a reversible capacity of 958.4 mAh g^−1^ after 100 cycles. The AAO separators exhibit the distinct advantage of enhancing the performance of LSBs while circumventing issues related to poor compatibility with lithium-metal cathodes and elevated interfacial resistance encountered in other solid-state batteries. In addition, Shi et al. developed a multifunctional interlayer combining AAO membrane’s aligned nanochannels with porous 1T MoS_2_ nanotubes (MoS_2_@AAO) [104]. Owing to the AAO channel’s positive surface and minimal curvature, coupled with 1T MoS_2_’s high conductivity, and “sulfiphilic” and “lithiophilic” characteristics, the engineered interlayer guarantees rapid ion/electron transport and efficient prevention of polysulfide shuttling, and ensures consistent and stable lithium deposition throughout cycling.

As mentioned above, transition metal oxides have strong polar surfaces and can effectively chemically adsorb polysulfides. In addition to transition metal oxides, Wang et al., for the first time, used binary transition metal oxides (BTMO) MnFe_2_O_4_ prepared by the coprecipitation method to modify separators, as shown in Figure 7c [105]. The battery with a MnFe_2_O_4_ mass ratio of 5% exhibited the best cycling performance, as shown in Figure 7d. BTMO has a strong polar surface and contains two kinds of metal ions and oxygen anions, which are able to form different chemical bonds with polysulfides. The dual-functional modified separator, adorned with MnFe_2_O_4_ and conductive AB, is capable of hindering polysulfide diffusion, thanks to the combined impact of efficient chemical adsorption and supplementary physical obstruction. The effects of PP and MFO-5% separators on limiting polysulfide migration are shown schematically in Figure 7e,f. Dissolved polysulfides readily interact with the lithium-metal anode through the pure PP separator, leading to active material degradation and cell deterioration. The bifunctional MFO-5% separator mitigates the shuttle effect more effectively than the PP separator. The batteries equipped with the modified separators demonstrated impressive rate capacities of 920 mAh g^−1^ at 1 C and 845 mAh g^−1^ at 2 C, with a minimal capacity decay of just 0.074% per cycle.

In addition, it is well known that perovskite-type oxides can assume a rather high concentration of anionic vacancies [106]. Wang et al. innovatively modified commercial polyethylene membranes (Celgard) for Li-S batteries by applying a layer of perovskite lithium lanthanum titanate/Super P composite (LLTO/SP). Separators coated with LLTO/SP successfully obstructed the infiltration of lithium polysulfides [107]. The schematic diagram of the LLTO@Li-S battery is shown in Figure 7g. The separator features a continuous dense coating on a Celgard membrane support, incorporating interconnected SP and LLTO particles. The rough surface of the S/CNT cathode ensures contact with the LLTO/SP layer through carbon nanotubes. This cell structure facilitates the chemisorption and electrochemical conversion of polysulfides on the separator, enhancing the specific capacity and cycling performance of LSBs. Compared to LSBs with uncoated separators (Pristine@Li-S), LLTO@Li-S batteries have significantly lower impedance and higher lithium-ion diffusion coefficient. The LLTO@Li-S battery, with a conventional sulfur loading of 2 mg/cm^2^, achieves an initial specific capacity of 1491 mAh g^−1^ at 0.1 C and maintains a specific capacity of 459 mAh g^−1^ after 1200 cycles at 1 C. Furthermore, Huang et al. designed a commercial membrane modified with perovskite-type LaFeO_3_ nanocages [108]. Utilizing LaFeO_3_ to modify the separator enables the LaFeO_3_ functional separator to efficiently contain polysulfides, reduce the shuttle effect, and enhance sulfur usage. Utilizing LaFeO_3_ functional separators, the battery demonstrates superior performance in terms of rate and extended cycle life.

## 3. Prospects and Outlooks

The improvement of lithium–sulfur battery separators has been widely studied. Among the modified materials mentioned above, carbon materials have good electrical conductivity, which can effectively promote the transmission of lithium ions and improve the charging and discharging efficiency and stability of the battery. Although carbon materials generally have good conductivity, certain specific types of carbon materials may have insufficient conductivity, affecting the performance of the battery. In practice, carbon materials often need to be combined with other functional materials to achieve better performance, which adds complexity and cost to the preparation process. Polymer materials have good ionic conductivity, which can facilitate the transport of lithium ions in batteries. However, some polymer materials may decompose or lose structural stability at high temperatures, affecting the safety performance of the battery. Metal compound materials usually have better chemical stability and can resist corrosion of the electrolyte. Compared to polymer materials, metal compound materials are usually heavier, which may increase the overall weight of the battery and reduce energy density. Some metal compound materials have low conductivity, potentially increasing internal battery resistance and affecting discharge performance. As lithium–sulfur battery technology continues to evolve, the performance requirements for battery separators are becoming increasingly strict.

LSBs are an important new battery technology with the advantages of high energy density and low cost. As a crucial element in LSBs, the separator directly affects their performance and safety. The development of new separator materials involves nanocomposites, porous separators, and functionalized polymers. The future trend is to design separators with multifunctional properties, such as lithium storage, blocking the growth of dendrites and enhancing the ion transport rate. As materials science and engineering technologies advance, innovative separators are set to enhance the efficiency of LSBs, encompassing their cycle life, energy density, and safety. Overall, the development of LSB separators is promising, and advances in new materials and engineering design will further promote the commercialization and large-scale application of LSB technology.

## Data Availability

No new data were created or analyzed in this study. Data sharing is not applicable to this article.
